# There’s a storm a‐coming: Ecological resilience and resistance to extreme weather events

**DOI:** 10.1002/ece3.6842

**Published:** 2020-10-01

**Authors:** Eric W. Neilson, Clayton T. Lamb, Sean M. Konkolics, Michael J. L. Peers, Yasmine N. Majchrzak, Darcy Doran‐Myers, Laura Garland, April Robin Martinig, Stan Boutin

**Affiliations:** ^1^ Biological Sciences University of Alberta Edmonton AB Canada; ^2^ Natural Resources Canada ‐ Canadian Forest Service Edmonton AB Canada

**Keywords:** climate change, disturbance, extreme weather events, resilience, resistance

## Abstract

Extreme weather events (EWEs) are expected to increase in stochasticity, frequency, and intensity due to climate change. Documented effects of EWEs, such as droughts, hurricanes, and temperature extremes, range from shifting community stable states to species extirpations. To date, little attention has been paid to how populations resist and/or recover from EWEs through compensatory (behavioral, demographic, or physiological) mechanisms; limiting the capacity to predict species responses to future changes in EWEs. Here, we systematically reviewed the global variation in species’ demographic responses, resistance to, and recovery from EWEs across weather types, species, and biogeographic regions. Through a literature review and meta‐analysis, we tested the prediction that population abundance and probability of persistence will decrease in populations after an EWE and how compensation affects that probability. Across 524 species population responses to EWEs reviewed (27 articles), we noted large variation in responses, such that, on average, the effect of EWEs on population demographics was not negative as predicted. The majority of species populations (80.4%) demonstrated compensatory mechanisms during events to reduce their deleterious effects. However, for populations that were negatively impacted, the demographic consequences were severe. Nearly 20% of the populations monitored experienced declines of over 50% after an EWE, and 6.8% of populations were extirpated. Population declines were reflected in a reduction in survival. Further, resilience was not common, as 80.0% of populations that declined did not recover to before EWE levels while monitored. However, average monitoring time was only two years with over a quarter of studies tracking recovery for less than the study species generation time. We conclude that EWEs have positive and negative impacts on species demography, and this varies by taxa. Species population recovery over short‐time intervals is rare, but long‐term studies are required to accurately assess species resilience to current and future events.

## Graphical abstract

Extreme weather events (EWEs) are expected to increase in stochasticity, frequency, and intensity as a result of climate change. Through a literature review and meta‐analysis, we tested the prediction that population abundance and probability of persistence will decrease in populations after an EWE. Across 524 species responses to EWEs reviewed (27 articles), we noted large variation in responses, such that, on average, the effect of EWEs on population demographics was not negative as predicted.

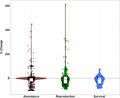

## 1 INTRODUCTION

The stochasticity, frequency and intensity of extreme weather events (EWEs) are predicted to increase due to climate change (Coumou & Rahmstorf, 2012; Easterling, 2000; Meehl & Tebaldi, 2004; Ummenhofer & Meehl, 2017). EWEs are defined as events outside the range of normal values or as rare within a statistical range at a particular place and time of year (Bailey & van de Pol, 2016; IPCC, 2001; Jentsch et al., 2007) and include droughts, storms such as hurricanes and tornadoes, floods, and extreme temperature. EWEs can have destructive effects on species, ecological communities, and ecosystems, (Easterling, 2000; Parmesan et al., 2000) and the projected increase in EWE frequency and intensity represents a concerning consequence of climate change for species persistence and biodiversity globally (Jentsch et al., 2007).

The likelihood that a species persists after exposure to a disturbance is a function of both its capacity to resist the deleterious effects of that disturbance, and its resilience—its ability to recover from any effects that occur (Holling, 1973; McKinney, 1997). Estimating population resistance provides insight into how immediately damaging a disturbance can be, but is insufficient for predicting persistence because populations may be able to rapidly recover from negative impacts (i.e., low resistance but high resilience). Life‐history pace and immigration may compensate for an insufficient ability to resist negative EWE effects, allowing populations to be largely unaffected in the long term (Barnthouse, 2004; Mutz et al., 2017). The assessment of both resilience and resistance is particularly important when the population is exposed to sequential disturbances because if the disturbance interval is shorter than the recovery interval, even a highly resistant population will decline over time (Fairman et al., 2016; Paine et al., 1998). Therefore, predicting the effect of increasing EWE frequency and intensity on species persistence requires an estimate of the magnitude and direction of the effect of a single EWE on population abundance, as well as the rate that populations recover.

Whereas steep population decline after an EWE are expected, resulting effects on population persistence are not always predictable due to compensatory (behavioral, demographic, or physiological) mechanisms by which a population may resist or recover from the effects of EWEs. A population or species may alter its spatial distribution or phenology, thereby reducing exposure to conditions outside its tolerance limits through behavioral mechanisms (Brown et al., 2016; Chen et al., 2011; Easterling, 2000; Parmesan et al., 2000). Demographically, when an EWE reduces population abundance but habitat remains, density‐dependent feedbacks in population growth may allow population recovery (Vandermeer et al., 1996). Species resistance and resilience to EWEs may also vary with specific traits that affect population growth rates, such as body size (Savage et al., 2004) and generation time, specifically shorter generation times should increase the rate of recovery (Paine et al., 1998). As a result, certain taxonomic groups may face differential risk of extirpation from such events.

Concern over the effect of EWEs on population persistence due to climate change has prompted a shift in climate research from trends to events (Easterling, 2000; Jentsch et al., 2007; Maxwell et al., 2018; Parmesan et al., 2000). However, because most research on EWEs has focused on the effect of single EWEs on single species or taxa, broad and reliable predictions for the effects on biodiversity are only possible through a review and meta‐analysis of species resistance and resilience after the passage of an EWE (Altwegg et al., 2017). Here, we examine the current knowledge of EWE effects on species persistence, and the vulnerability of species to changes in EWE frequency or intensity, by performing a meta‐analysis of reported effects of EWEs on population resistance and resilience. Our analysis defined population resistance as the change in population abundance, survival, and reproduction after an EWE. We estimated population resilience by calculating the probability that a population, which declined due to an EWE, would return to pre‐EWE abundance during the time it was monitored. Finally, we examined how resilience depended on species traits and the magnitude of the EWE effect on the population.

## 2 MATERIALS AND METHODS

We queried the Web of Science on 16 October 2017 using the search terms “extreme weather event” and “species” and on 21 January 2018 for search terms “extreme weather event” and “populations.” Additionally, we queried Scopus on 21 January 2018 using the same search terms. Whereas we did not conduct an exhaustive search, we consider our search terms to be unbiased and representative of the literature. These searches resulted in 336 peer‐reviewed, scientific articles between 1992 and 2017. We removed studies in which the authors did not explicitly state that they measured a demographic response to a EWE and those studies reporting the results of experiments. We further removed studies that did not report the effects of these events on species abundance or demography and could not be calculated as a percent change.

Our approach was to isolate the effect of a single EWE, on a single population and therefore did not include cumulative, indirect or repeat exposure effects, nor interactive effects from other sources such as human disturbance. From each reviewed article, we considered separately the responses to the EWE for each species or species population (hereafter; population) that was assessed in the study. We also included studies that measured responses for groups of related organisms. We included changes in abundance, survival, and reproduction for each population and recorded the type, magnitude, and direction of response of these demographic parameters to the event. Whenever possible, we recorded the reported percent change in the demographic parameter. However, when studies reported the population metric before and after an EWE, or from an affected area and a control area, we calculated the percent change as:

$$\lambda =\frac{\left(N_{t}‐N_{t+1}\right)}{N_{t}}$$

where λ is the percent change in a metric, *N_t_* is the control or the value of the metric post‐EWE, and *N_t_*_+1_ is the treatment or value of the metric post‐EWE. We also recorded the geographic location of the EWE, the taxonomic classification of the species monitored, and the type of weather event. We grouped events into four distinct categories: (a) storms, which included hurricanes, cyclones, and other listed storm events, (b) droughts, (c) floods, and (d) temperature extremes, which included extreme variations in weather such as heat waves. We relied on author descriptions of the EWE to determine classification. We further restricted our dataset to more accurately isolate the effects of EWEs on population demography. To ensure events included were a true extreme weather event given the background weather variation of the study area, we excluded studies that did not include a measure of intensity context, that is, whether EWE of interest was compared to background variation. This resulted in a final 27 studies that examined the demographic responses to extreme weather from a range of biogeographical regions. We measured and accounted for inconsistency in our review data collection by calculating a repeatability score for a subset of papers (see Supplemental Material).

To examine the magnitude and direction of population demographic responses to EWEs, we calculated the mean and 95% confidence intervals (CI) of the response percentage change in each of our three population metrics of interest by bootstrap resampling of the distribution of reported values 10,000 times. For responses in abundance, we recorded whether the paper identified any compensation in the population response to the EWE. Compensation was defined as any means by which the population reduced the effects of the EWE and was categorized as demographic (increased reproduction or survival), physiological, or behavioral. For populations that declined, we measured the difference in percent change in abundance between populations that compensated for the EWE and those that did not. We recalculated the bootstrap mean response of abundance to an EWE for those population that declined to examine the average decline for population that were negatively affected by EWEs. We also recorded whether the researchers monitored the population for recovery after the event. In studies that monitored recovery, we recorded the time (in days) that recovery was monitored, and whether the population recovered to before EWE abundance.

To examine mechanisms driving population resistance to EWEs, and their propensity to recover, we gathered additional information on these species from external databases. Age of female maturity (included as a surrogate for generation time) and body size data for species were obtained from online databases (Kleyer et al., 2008; Myhrvold et al., 2015). For papers that measured the response of EWEs above the species level, we used an average for the nearest taxonomic classification from these databases to those used in the paper. For species not included in these databases, we found this information from peer‐reviewed literature and expert opinion. We were interested in three questions: how do species attributes influence the (a) probability of population decline or increase after a EWE? (b) magnitude of decline post‐EWE? and (c) probability of recovering to the pre‐ EWE abundance? For question 1, we fit general linear models predicting the probability of decline to the subset of populations that measured compensation (138 populations). For question 2, we fit linear regression models predicting magnitude of decline to the subset of populations that declined and measured compensation (90 populations). For question 3, we fit logistic regression models predicting the probability of recovery to the same data, subset to records that also measured recovery (82 populations). For each question, we compared a set of candidate models and made our inference from the model‐averaged coefficients from the top model, ranked using AIC weight (Appendix 1) (Burnham & Anderson, 2002). For questions 1 and 2, our candidate models regressed our response variable against all combinations of the following variables; species group (either plants, vertebrates, or invertebrates), presence of compensation, the type of EWE experienced. For question 3, candidate models additionally included body size, age of maturity, the duration post‐EWE that recovery was monitored, and the magnitude of decline caused by the EWE.

## 3 RESULTS

### 3.1 Magnitude of effect of EWEs on species demography and population persistence

**FIGURE 1:**
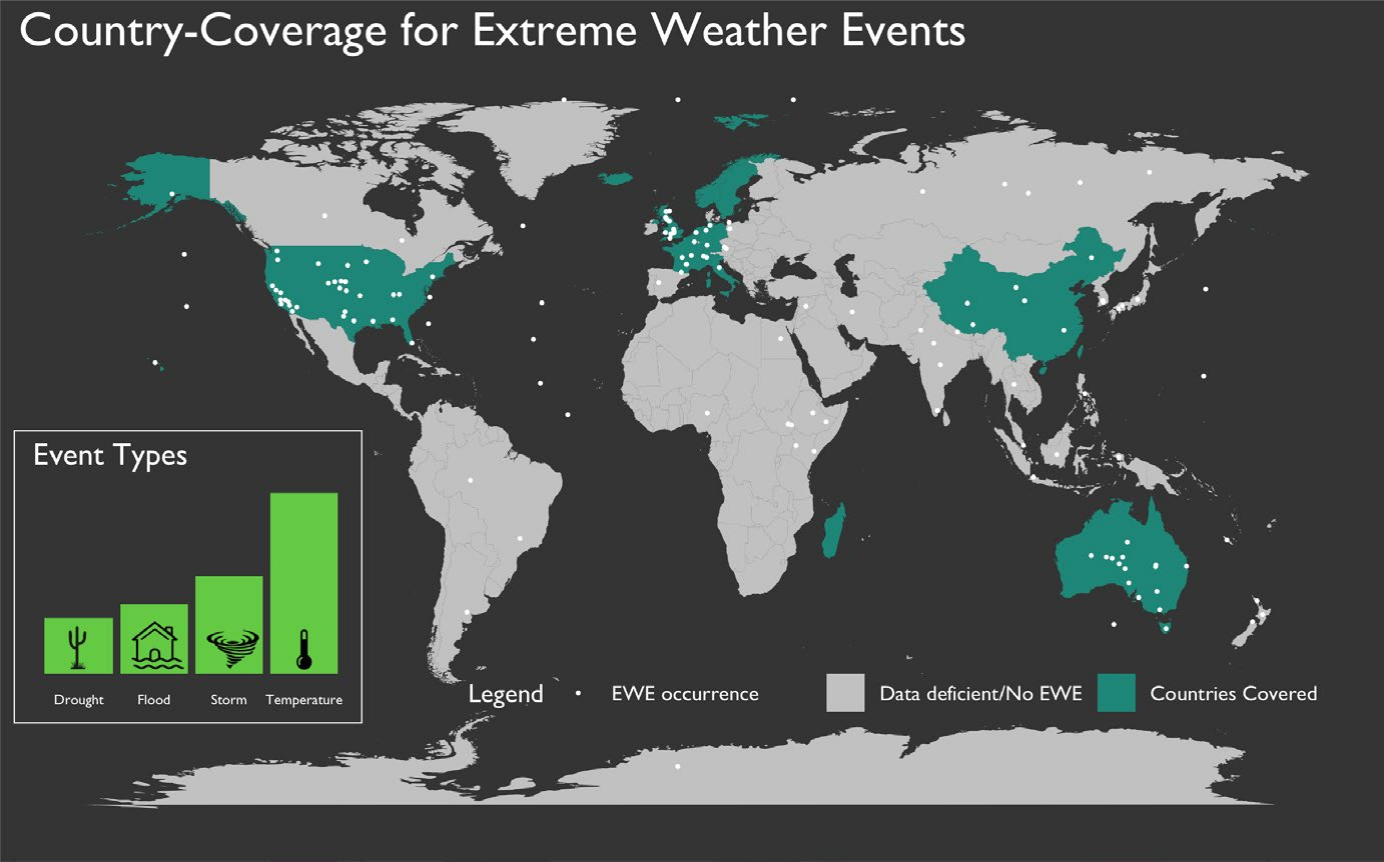
Location of EWEs (white dots) as reported by the American Meteorological Society (Herring et al., 2019). Countries colored in green contain the locations of the demographic responses to EWE in our review that reported the context of the EWE intensity and the demographic responses. Countries colored in gray did not have any reports of demographic responses to EWE that included the context of the EWE intensity and the demographic response. Inset map displays the frequency distribution of each of the EWE categories in our review

We retained 27 studies that compared the change in population abundance, survival, or reproduction after exposure to an EWE, and further included the context of the event to background climate conditions. These 27 studies included 524 separate reports of species population or community responses to EWEs over a wide geographic distribution, with effects measured from all continents, and a roughly even number of EWE types (Figure 1). Avian species dominated these 524 records, with 312 avian, 144 angiosperms, 20 arthropods, 17 mammals, 10 reptile records, and amphibians, chlorophytes, fish, plants, mollusks, and nematodes encompassing the remaining 21 records.

**FIGURE 2:**
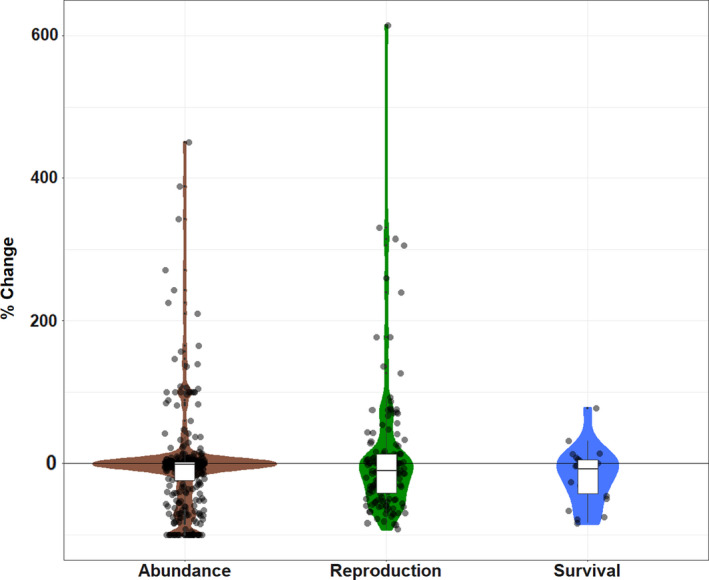
The effect of extreme weather events on population abundance, reproduction, and survival summarized from 524 records from 27 papers. Colored areas are violin plots estimated with a Gaussian kernel. Boxplots represent the median and interquartile range of the percent changes for each response. The −100% value on the y‐axis represents extirpation of the population

The majority of population responses to EWEs reported changes in abundance (352 records, 67.2% of total). These 352 records encompassed 108 different species populations or species groups. The majority of these species were <0.5 kg, and all were <11 kg, likely reflecting the difficulty of attaining demographic information for large, wide‐ranging species (Lamb et al., 2019). The bootstrap mean change in population abundance was −4.96% and did not differ statistically from 0 (95% CI −11.4, 2.1; Figure 2). Overall, 28.7% percent of populations declined by 1%–25%, 13.6% by 25%–75%, 4.3% by 75%–99%, and 24 study populations were completely extirpated (6.8%). However, 21.0% of populations increased in abundance by 1%–50%, 8.2% of populations increase >50%, and 5.4% of populations were unaffected by the event (0% change).

**FIGURE 3:**
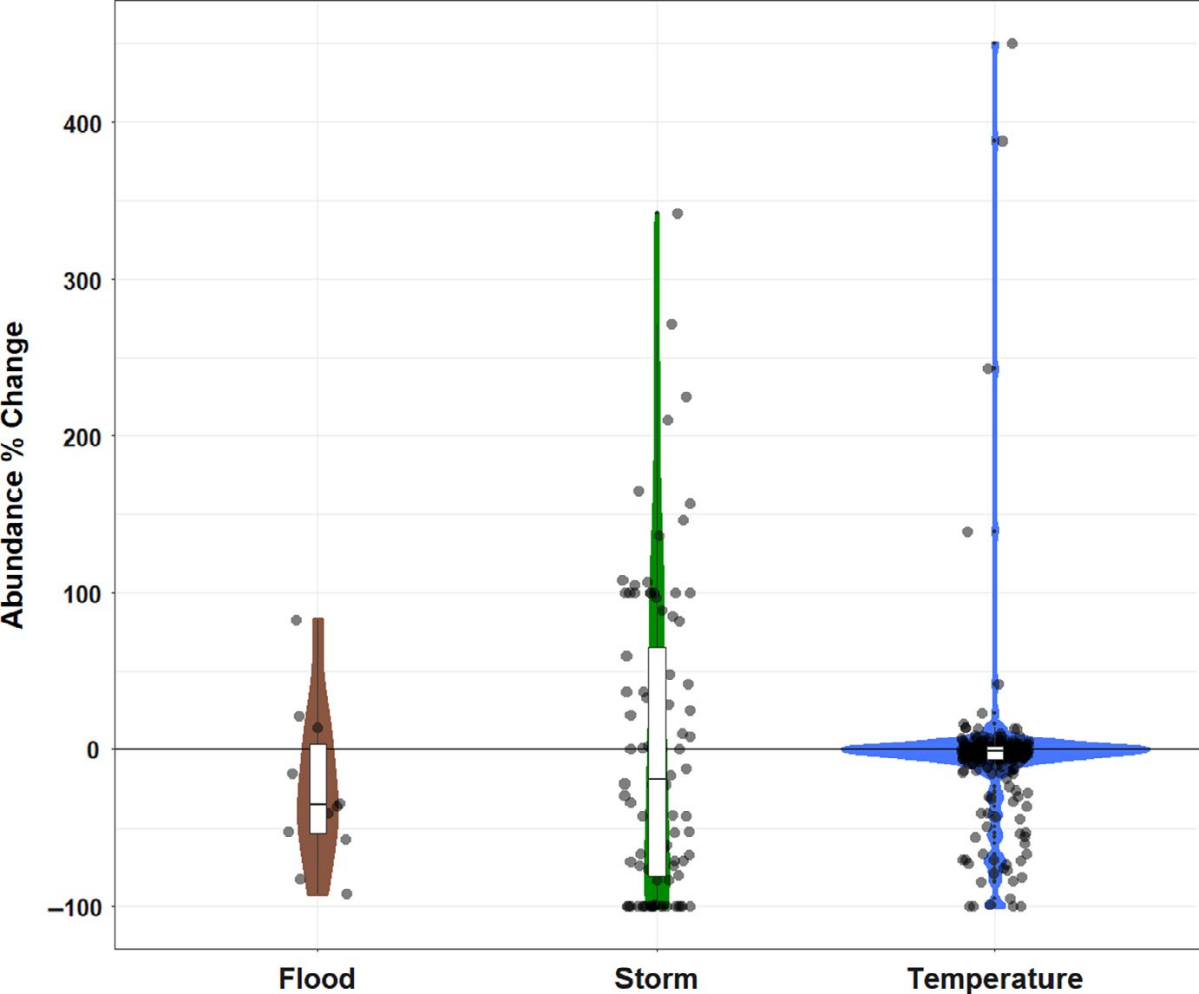
The effect of three different categories of extreme weather events on population abundance summarized from 352 records from 14 papers. Colored areas are violin plots estimated with a Gaussian kernel. Boxplots represent the median and interquartile range of the abundance changes for each EWE type. The −100% value on the y‐axis represents extirpation of the population

Of the studies that measured changes in abundance, following a weather event defined by the authors as extreme in the context of the background weather variation, the majority (69.9%) examined the effects of temperature extremes, which elicited only weak effects on population abundance (Fig 3, −5.24% (95% CI −10.8, 1.6). On average, floods caused more severe population declines (−24.2% (95% CI −49.1, 2.34) than temperature or storms (−0.74%, 95% CI −19.6, 19.1) (Figure 3). We only reviewed two records of the effect of droughts on abundance, in which one population did not respond to the drought and the other was extirpated.

In addition to the 352 records of abundance responses to EWEs in the 27 studies of demographic responses, we reviewed 149 reproduction and 23 survival responses. The mean change in reproduction post‐EWE was 4.84% (95% CI −8.44, 20.5; Figure 2) and did not differ statistically from 0. The mean change in survival post‐EWE was −17.8% (95% CI −33.0,‐2.47; Figure 2) and differed significantly from 0.

**FIGURE 4:**
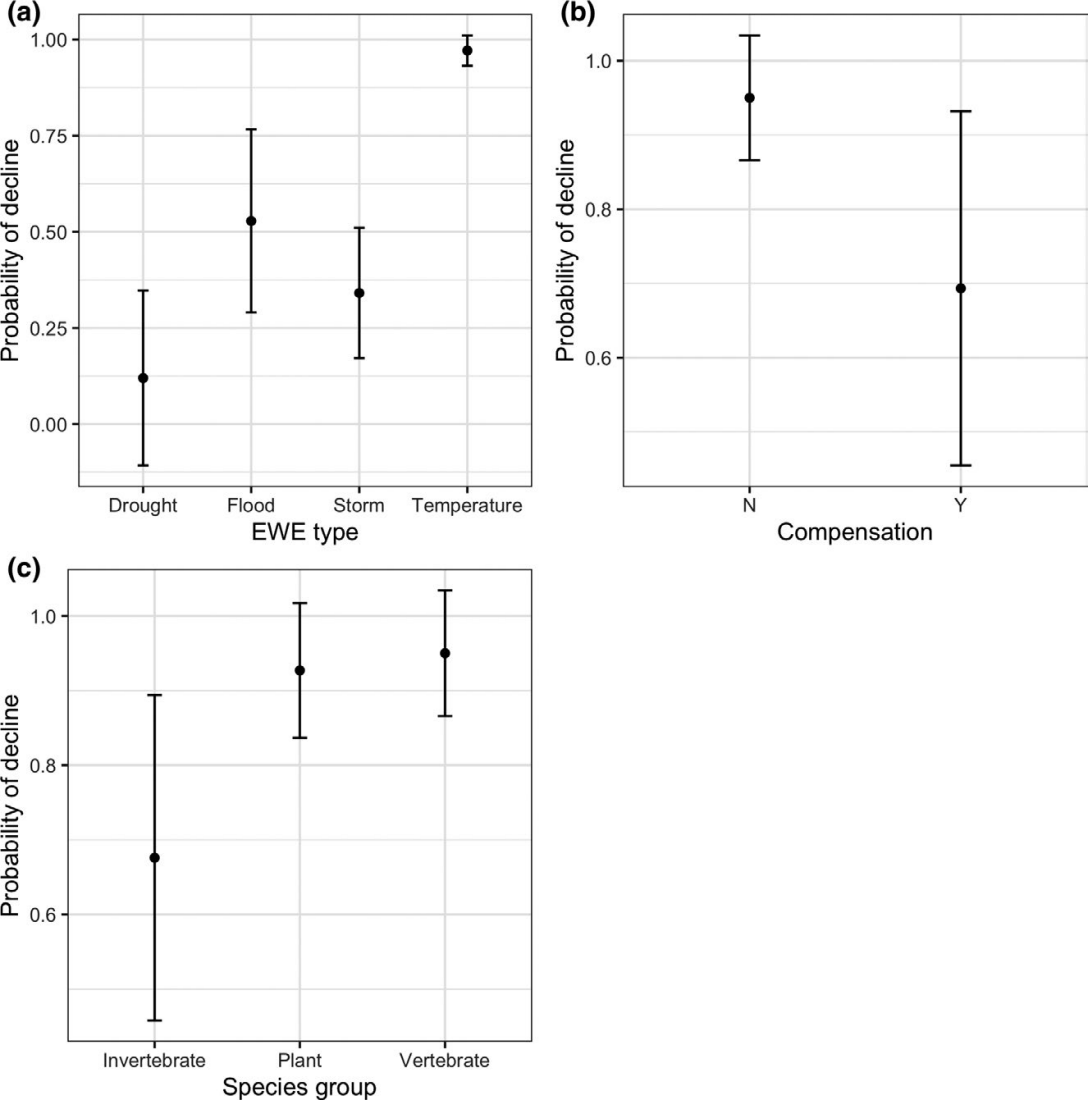
Model predicted probabilities of populations decline after the passage of an extreme weather event (EWE) as functions of species group, type of EWE and whether the population compensated for the EWE. Compensation was defined as any means by which the population reduced the effects of the EWE and was categorized as either demographic (increased reproduction or survival), physiological, or behavioral. Effects were estimated using logistic regression. We selected models within two ΔAIC of the top ranking model and averaged the coefficients using AIC weights. Error bars represent predicted response standard errors. 138 records were used for this analysis

**FIGURE 5:**
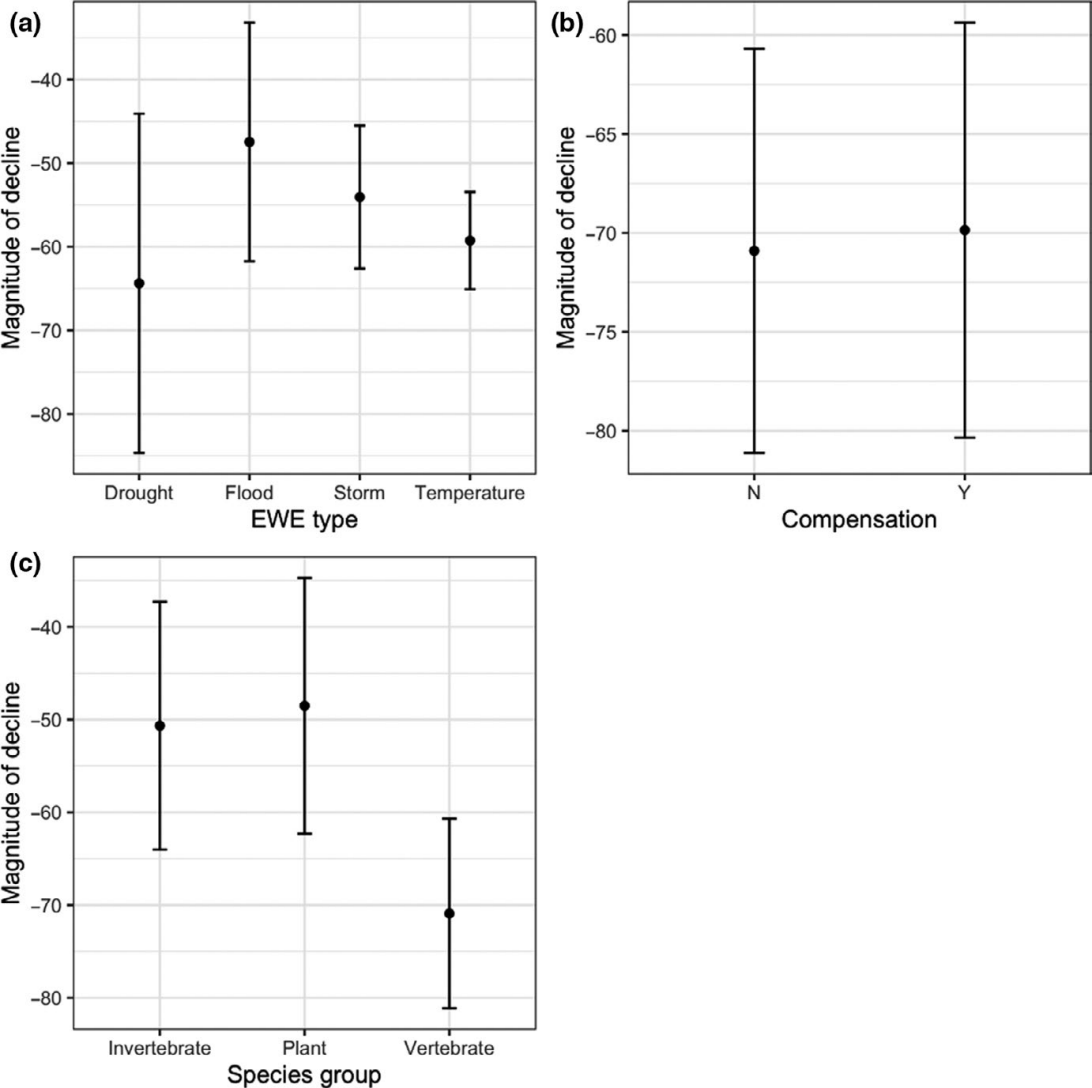
Model predicted linear coefficients of population decline after the passage of an extreme weather event as functions of species group, type of EWE, and whether the population compensated for the EWE. Compensation was defined as any way the population reduced the effects of the EWE and was categorized as either demographic (increased reproduction or survival), physiological, or behavioral. Effects were estimated using linear regression. We selected models within two ΔAIC of the top ranking model and averaged the coefficients using AIC weights. Error bars represent predicted response standard errors. 90 records were used for this analysis

Model selection (Appendix 1) suggested that vertebrates were most likely to decline, followed by plants and invertebrates. Modeling demonstrated the importance of controlling for the type of species and compensation. Our top abundance change model found that EWEs based on temperature were the most likely to cause a decline (Figure 4), in contrast to abundance changes among records that did not measure compensation. For the populations that did decline (90), the top predictor of decline magnitude was species group, with vertebrate populations experiencing the steepest declines, followed by invertebrates, then plants (Figure 5).

### 3.2 Compensation and species resilience

Of the studies where compensation was quantifiable, the majority of populations (80.4% out of 138 populations) did compensate for the damaging effect of EWEs, such that the effect of EWEs on population abundance was reduced by 86.9%. Populations compensated using behavioral (e.g., altering space use and range displacement), physiological (e.g., immune function and fat stores), or demographic (e.g., immigration) responses, but the majority of compensation reported was demographic (90.1%).

**FIGURE 6:**
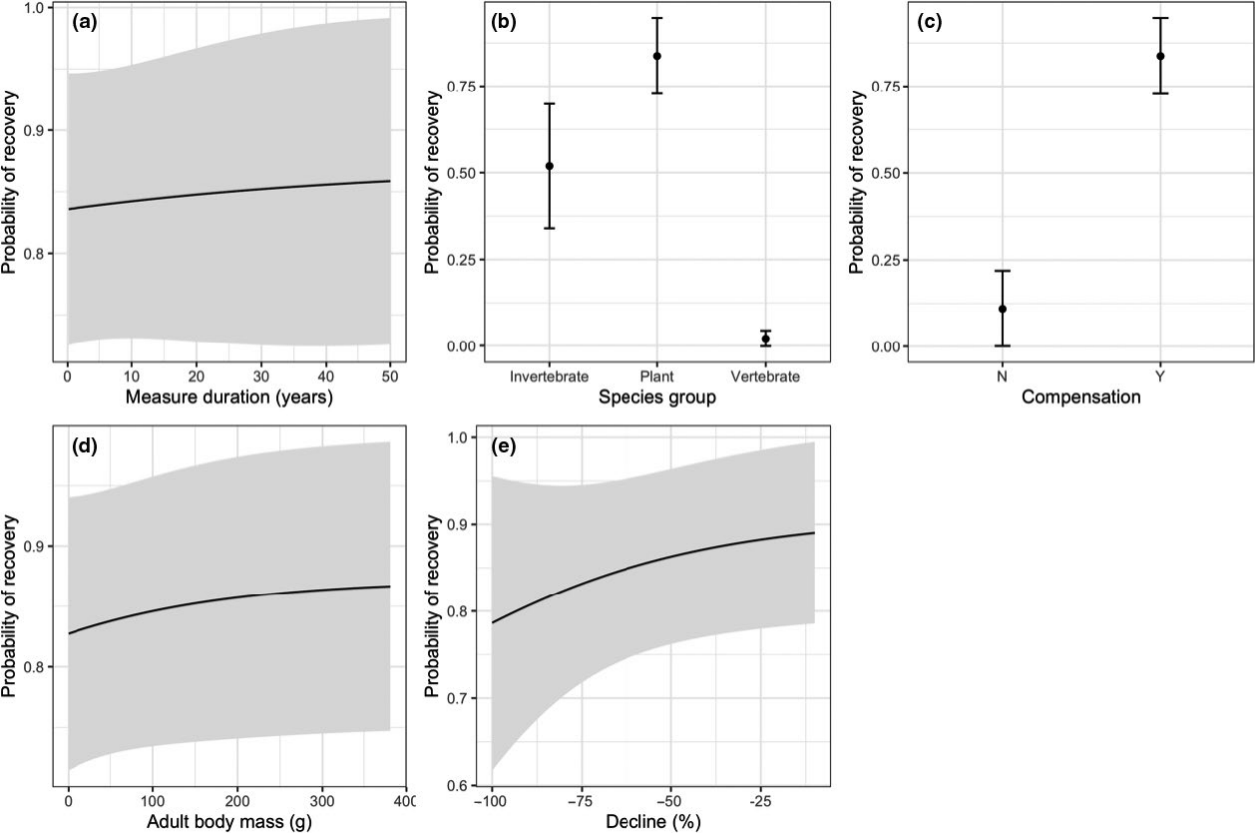
Model predicted probabilities of population recovery after the abundance decline due to the passage of an extreme weather event (EWE) as functions of body mass of the species, the duration the population was monitored for a recovery (Measure duration), whether the species exhibited any compensation in response to the EWE (Compensation), species group, the species; female age of maturity and the magnitude of the decline caused by the EWE (decline (%)). Compensation was defined as any means by which the population reduced the effects of the EWE and was categorized as either demographic (increased reproduction or survival), physiological, or behavioral. Effects were estimated using logistic regression. We selected models within two delta AIC of a top ranking model and averaged the coefficients using AIC weights. Error bars or limits (represented by the gray area) represent the predicted response standard errors. 82 records were used for this analysis

Despite the high percentage of populations that compensated for the effects of EWEs, our review did not reveal strong population recovery. When a population experienced a decline (59.4% of records), the majority (80.0% of 90 records in which recovery was monitored) of populations did not recover to pre‐EWE abundance prior to the conclusion of the study. When populations declined by more than 90%, only 6.8% were able to recover. However, the mean monitoring duration of populations post‐EWE was generally short (2 years) and was shorter than the age to female maturity in 28% of cases. Model selection (Appendix 1) suggested that the duration of monitoring post‐EWE influenced the probability of detecting recovery, and populations that compensated for the effects of the EWE or that had less severe declines were more likely to recover after they declined (Figure 6). Plants and larger bodied fauna were more likely to recover than either vertebrate or invertebrate populations (Figure 6).

## 4 DISCUSSION

Our goal was to evaluate the demographic resistance and resilience of species to various extreme weather events, addressing earlier calls (Jentsch et al., 2007) to gain insight on how the projected increase in EWE intensity and frequency will impact biodiversity. We found that the effects of EWEs on demography were not negative across all species, as many exhibited a high degree of resistance. Compensation in either behavior or demography allowed for the majority of populations to experience only minor changes in abundance. There was, however, large variation in population responses, as some populations’ experienced drastic increases in abundance while a subset declined to local extirpation. Of the populations that declined, only 20% were able to recover to their previous abundance, and the probability of recovery decreased with the magnitude of decline. Our results suggest that whereas extreme weather does not always cause population declines, some populations are vulnerable to EWEs. We further discuss potential reasons for the variation in declines and how these factors may influence species persistence under future climate landscapes.

### 4.1 Population responses to extreme events

Contrary to our predictions and the overall expectations from the conservation literature (Easterling, 2000; Parmesan et al., 2000), we found that species are largely resistant to extreme weather events. The majority (54.8%) of EWEs elicited population responses with a magnitude of <10%, indicating that populations are often unaffected. Physiological and behavioral compensation after EWEs suggests that species are adapted to the occurrence of such events where they occur (Hoffmann & Sgró, 2011). The demographic compensation revealed by our review suggests that density‐dependent feedback increases population growth post‐EWE (Defeo & McLachlan, 2005). Such feedback requires that the disturbance did not reduce all populations equally (Vandermeer et al., 1996) so that resources, such as habitat, remain for increased recruitment. However, we do not suggest that EWEs are of no concern for species and/or population persistence. Nearly 60% of the populations declined in abundance, with 32.5% of these populations declining more than 50%, and 6.8% of populations being extirpated. When populations declined, the average change in abundance was −33.3% (95% CI −38.0, −28.5), demonstrating that although on average EWEs may have minimal effect, species that are not resistant suffer large declines. Our results further indicate that the driver of these negative effects is through survival, as across all populations survival was reduced by 17.8% during an EWE, while reproduction was largely unaffected or increased.

We found that vertebrates are more vulnerable to extreme weather events than other taxonomic groups. Vertebrate populations were more likely to decline after an EWE than average, and if they declined, had the largest magnitude of decline relative to other taxa (Figures 4 and 5). Moreover, fewer vertebrates recovered post‐EWE (Figure 6), in comparison with the other taxa. This result is expected given vertebrates are larger bodied animals with longer generation times (Prugh et al., 2018), and conservation biologists should focus on such species if assessing risk of future EWEs on population persistence (Cardillo et al., 2005; Purvis et al., 2000). However, initial declines in vertebrates such as birds and larger mammals may be exaggerated, due to the increased movement ability and a propensity to spatially avoid these events (Field et al., 2019; Kindvall, 1995; Zhang et al., 2016).

True recovery success for vertebrates may also be higher than reported here, as monitoring durations were often shorter than their generation time, and recovery through demographic means would not be captured (Denney et al., 2002). In general, invertebrates were more resistant to EWEs and had a higher likelihood to recover post‐EWE. The propensity for invertebrates to recover relative to vertebrates may be an artifact of monitoring duration, as after EWE monitoring was on average longer than invertebrate generation time.

Populations were least resistant to flood events, while both storms and temperature extremes did not significantly influence population demography. These results suggest species may be highly adapted for resisting storm events or temperature extremes in systems where they evolved (Hoffmann & Sgró, 2011; White, 1979). We only examined two instances of population responses to drought in our review, although in one instance this led to population extirpation. Drought may have significant impact on populations, as severe drought can lead to numerous physiological impacts on population survival and growth rates (Chesson & Huntly, 1997; de Jeu et al., 2013). More recently, (Prugh et al., 2018) found large demographic effects of long‐term drought across a wide range of taxa in the semi‐arid grasslands of California.

### 4.2 Predicting population vulnerability to future EWE

Species ability to compensate or remain resistant to EWEs will depend on whether future intensity or frequency of EWEs remains at levels under which adaptive responses arose (White, 1979). However, mounting evidence suggests that EWEs will increase in their frequency, intensity, and/or duration in coming decades (Russo et al., 2016; Schär et al., 2004; Ummenhofer & Meehl, 2017). More intense EWEs could cause declines in populations that are currently resistant, and more frequent EWEs can consistently decrease population abundance prior to their full recovery (Fairman et al., 2016; Paine et al., 1998). We attempted to examine these relationships through a meta‐analysis to forecast how changes in EWE frequency and intensity will impact species. Our analysis demonstrates that short‐term recovery is rare for most populations, especially vertebrates, and drastic increases in EWE frequency will strongly limit population resilience. Most studies, however, measured recovery for only short‐time periods (91% <2 years) that were below the generation time for over a quarter of the populations measured. Furthermore, the likelihood of recovery was slightly less likely with shorter monitoring durations (Figure 6), and therefore, the published studies in our review did not generally provide adequate estimates of resilience, which was likely biased low.

In order to examine species vulnerability to future EWEs, monitoring long term for recovery is critical to provide accurate assessments of resilience rates, as well as time until recovery (see Bailey & van de Pol, 2016). In addition, to understand whether increased EWE intensity or duration will alter species population resistance or resilience, we need to relate intensity with the magnitude of the effect on abundance and the propensity to recover. Species resistance may deteriorate under more extreme events (Parmesan et al., 2000) or when events proceed for longer durations (Prugh et al., 2018). However, these data are currently limited in the literature, as only 16% of studies in our initial review provided context of the intensity of the weather event.

To improve our predictive capacity of population responses to changes in event frequency and intensity, we recommend the following: (a) provide a measure of intensity and duration of the event based on standardized definitions (see Wright et al., 2015), (b) monitor populations prior to the event to appropriately assess whether demographic changes are larger than typical variation (see Ujvari et al. 2016), and (c) monitor populations after the event for longer durations that will capture recovery based on generation times of the study species (see Altwegg et al., 2006). Ideally, studies would monitor several species in the community simultaneously to examine more complex dynamics such as whether negative effects are caused by direct stress from the event or indirectly through changes in competition and predator–prey dynamics (Ujvari et al. 2016; Prugh et al., 2018). Further, species vulnerability to EWEs will vary over time if species adapt to extreme events. Adaptation will occur when heritable traits increase an individual's fitness after an EWE (Hoffmann & Sgró, 2011; White, 1979). We therefore suggest that measures of demography be compared against behavior, morphology, and other traits measured among individuals after the passage of an EWE to better understanding the potential for climate adaptation. Following these guidelines will improve our capacity to examine the resistance of populations to increased EWE intensity, and long‐term recovery potential under increased EWE frequency. Although such studies are technically challenging, they will greatly improve our predictive capacity of species vulnerability to climate change.

## 5 CONCLUSION

We defined species vulnerability to EWEs as the combination of the ability to resist and recover (resilience) from population declines after an EWE. By summarizing both the average decline after an EWE and the means by which that decline was mitigated, we estimated that, on average, populations resisted EWE effects. However, a substantial portion of populations responded negatively to these events, particularly vertebrates, and 24 populations were locally extirpated. Resilience after decline was not common, and however, most studies did not monitor recovery for long enough durations post‐EWE to accurately estimate resilience rates. We conclude that EWE can have negative impact on a subset of populations, but more long‐term studies are required to determine whether an increase in the frequency or magnitude of EWEs will increase species vulnerability. However, even with some population declines, EWEs have fewer negative effects on population abundance than previously thought. We do not suggest EWE’s are universally unimportant, but rather shed light on the variation in species response. An understanding of how EWE and the traits of the species concerned interact to then result in a negative demographic outcome is needed. Studies with more emphasis on before and after monitoring of populations can greatly improve the predictive power of climate modeling and projected species persistence in the future.

## CONFLICT OF INTERESTS

The authors declare no competing interests.

## AUTHOR CONTRIBUTION

**Eric W. Neilson:** Conceptualization (equal); Formal analysis (lead); Investigation (lead); Resources (equal); Writing‐original draft (lead); Writing‐review & editing (lead). **Clayton T. Lamb:** Conceptualization (equal); Formal analysis (lead); Investigation (lead); Resources (equal); Visualization (lead); Writing‐original draft (equal); Writing‐review & editing (equal). **Sean M. Konkolics:** Conceptualization (equal); Investigation (equal); Resources (equal); Writing‐original draft (equal); Writing‐review & editing (equal). **Michael J. L. Peers:** Conceptualization (equal); Investigation (equal); Resources (equal); Writing‐original draft (equal); Writing‐review & editing (equal). **Yasmine N. Majchrzak:** Conceptualization (equal); Investigation (equal); Resources (equal); Writing‐original draft (equal); Writing‐review & editing (equal). **Darcy Doran‐Meyers:** Conceptualization (equal); Investigation (equal); Resources (equal); Writing‐original draft (equal); Writing‐review & editing (equal). **Laura Garland:** Conceptualization (equal); Investigation (equal); Resources (equal); Writing‐original draft (equal); Writing‐review & editing (equal). **April Robin Martinig:** Conceptualization (equal); Investigation (equal); Resources (equal); Writing‐original draft (equal); Writing‐review & editing (equal). **Stan Boutin:** Conceptualization (equal); Investigation (equal); Supervision (lead); Writing‐review & editing (supporting).

## Supporting information

Supplementary Material

## Data Availability

The data we used for our meta‐analysis are available at https://doi.org/10.5061/dryad.c59zw3r58.
