# Supplementary material for: There’s a storm a‐coming: Ecological resilience and resistance to extreme weather events
Source: Ecol Evol. 2020 Oct 1;10(21):12147–56. doi: 10.1002/ece3.6842 (PMC7664005; doi:10.1002/ece3.6842)
Supplement: Supplementary file 1 — Supplementary Material [file ECE3-10-12147-s001.docx]

# Supplementary

## Review repeatability

We had alternate reviewers revisit 30% of papers, with a focus on those with < 5 rows, to ensure our data collection was repeatable. We repeated data collection on a subset of columns, to which our inference was most sensitive, including EWE type, effect size and direction, compensation and recovery. For each of these columns we created a repeatability score for each column (the percent of matching records between the original and revisited data). Columns that failed to produce a repeatability score of > 85 in our initial sub-sample, were revisited across all data (entire sample) by an additional reviewer and any discrepancies were discussed and revised based on group agreement.

## Review Database

We will publish, as a data table, the metrics collected for each species and EWE from each paper in our review.

*Model Selection*

| **Table AI. 1** Model selection values for selection among logistic regression models predicting the probability of species population decline after a EWE. Covariates include species group (sp.group2), a boolean indicating whether compensation occurred during or after the EWE, and EWE type (Type_Lump). | | | | | | | | |
| --- | --- | --- | --- | --- | --- | --- | --- | --- |
| (Intercept) | Compensation | sp.grouped2 | Type_Lump | df | logLik | AICc | delta | weight |
| -1.68 | + | + | + | 7 | -70.56 | 155.98 | 0.00 | 0.75 |
| 0.00 | + | NA | + | 5 | -74.12 | 158.70 | 2.72 | 0.19 |
| 0.00 | NA | NA | + | 4 | -76.96 | 162.23 | 6.25 | 0.03 |
| -1.01 | NA | + | + | 6 | -74.97 | 162.59 | 6.61 | 0.03 |
| 2.08 | + | NA | NA | 2 | -84.36 | 172.81 | 16.83 | 0.00 |
| 2.17 | + | + | NA | 4 | -82.86 | 174.01 | 18.03 | 0.00 |
| 1.22 | NA | + | NA | 3 | -85.90 | 177.99 | 22.01 | 0.00 |
| 0.63 | NA | NA | NA | 1 | -89.16 | 180.35 | 24.37 | 0.00 |

| **Table AI. 2** Model selection values for selection among linear regression models predicting the magnitude of species population decline after a EWE. Covariates include species group (sp.group2), a boolean indicating whether compensation occurred during or after the EWE, and EWE type (Type_Lump). | | | | | | | | |
| --- | --- | --- | --- | --- | --- | --- | --- | --- |
| (Intercept) | Compensation | sp.grouped2 | Type_Lump | df | logLik | AICc | delta | weight |
| -59.82 | NA | + | NA | 4 | -418.58 | 845.63 | 0.00 | 0.37 |
| -76.20 | NA | + | + | 7 | -415.31 | 845.98 | 0.35 | 0.31 |
| -61.08 | + | + | NA | 5 | -418.51 | 847.73 | 2.10 | 0.13 |
| -75.60 | + | + | + | 8 | -414.99 | 847.77 | 2.14 | 0.13 |
| -100.00 | NA | NA | + | 5 | -419.81 | 850.33 | 4.70 | 0.03 |
| -68.74 | NA | NA | NA | 2 | -423.73 | 851.60 | 5.97 | 0.02 |
| -100.00 | + | NA | + | 6 | -419.66 | 852.33 | 6.70 | 0.01 |
| -66.49 | + | NA | NA | 3 | -423.61 | 853.51 | 7.88 | 0.01 |

| **Table AI. 3** Model selection values for selection among logistic regression models predicting the probability of recovery among species populations that declined after a EWE. Covariates include the body mass of the species (A Body Mass, in grams), the duration the population was monitored for a recovery (M Dur, in days), whether the species exhibited any compensation in response to the EWE (Comp.), species group (sp.group2), female age of maturity (Fem Dur), and the magnitude of the decline caused by the EWE (Effect). | | | | | | | | | | | | |
| --- | --- | --- | --- | --- | --- | --- | --- | --- | --- | --- | --- | --- |
| (Intercept) | A. Body Mass | Comp. | Effect | Fem. Mat | M. Dur | sp.grouped2 | Type | df | logLik | AICc | delta | weight |
| -3.55 | NA | + | NA | NA | NA | + | NA | 4.00 | -19.22 | 46.96 | 16.57 | 0.30 |
| -2.34 | NA | + | 0.02 | NA | NA | + | NA | 5.00 | -18.42 | 47.63 | 17.24 | 0.21 |
| -3.55 | 0.01 | + | NA | NA | NA | + | NA | 5.00 | -18.95 | 48.69 | 18.29 | 0.13 |
| -3.59 | NA | + | NA | NA | 0.00 | + | NA | 5.00 | -19.17 | 49.13 | 18.73 | 0.10 |
| -2.24 | 0.01 | + | 0.03 | NA | NA | + | NA | 6.00 | -18.07 | 49.27 | 18.87 | 0.09 |
| -2.37 | NA | + | 0.02 | NA | 0.00 | + | NA | 6.00 | -18.37 | 49.85 | 19.45 | 0.07 |
| -3.43 | 0.02 | + | NA | NA | 0.00 | + | NA | 6.00 | -18.75 | 50.61 | 20.21 | 0.05 |
| -1.93 | 0.02 | + | 0.03 | NA | 0.00 | + | NA | 7.00 | -17.72 | 50.96 | 20.56 | 0.04 |
| -0.20 | NA | NA | NA | -0.01 | NA | + | NA | 4.00 | -25.55 | 59.62 | 29.22 | 0.00 |
| -0.09 | 0.01 | NA | NA | -0.01 | NA | + | NA | 5.00 | -24.83 | 60.45 | 30.05 | 0.00 |
| -0.79 | NA | NA | NA | NA | NA | + | NA | 3.00 | -27.16 | 60.63 | 30.24 | 0.00 |
| 0.68 | NA | NA | 0.02 | -0.01 | NA | + | NA | 5.00 | -25.06 | 60.91 | 30.51 | 0.00 |
| -0.13 | NA | NA | NA | -0.02 | 0.00 | + | NA | 5.00 | -25.12 | 61.04 | 30.64 | 0.00 |
| -19.57 | NA | NA | NA | NA | NA | NA | + | 4.00 | -26.31 | 61.14 | 30.75 | 0.00 |
| -17.59 | NA | NA | 0.02 | NA | NA | NA | + | 5.00 | -25.38 | 61.56 | 31.16 | 0.00 |
| 0.10 | NA | NA | 0.02 | NA | NA | + | NA | 4.00 | -26.59 | 61.70 | 31.31 | 0.00 |
| 0.81 | 0.01 | NA | 0.02 | -0.01 | NA | + | NA | 6.00 | -24.33 | 61.79 | 31.39 | 0.00 |
| -19.50 | NA | NA | NA | NA | 0.00 | NA | + | 5.00 | -25.53 | 61.84 | 31.44 | 0.00 |
| -19.23 | -0.01 | NA | NA | NA | NA | NA | + | 5.00 | -25.67 | 62.14 | 31.74 | 0.00 |
| -0.79 | 0.01 | NA | NA | NA | NA | + | NA | 4.00 | -26.92 | 62.36 | 31.96 | 0.00 |
| 0.75 | NA | NA | 0.02 | -0.02 | 0.00 | + | NA | 6.00 | -24.64 | 62.40 | 32.00 | 0.00 |
| -20.38 | NA | NA | NA | 0.00 | NA | NA | + | 5.00 | -25.82 | 62.43 | 32.04 | 0.00 |
| -19.32 | NA | NA | 0.02 | 0.00 | NA | NA | + | 6.00 | -24.74 | 62.61 | 32.21 | 0.00 |
| -17.70 | NA | NA | 0.02 | NA | 0.00 | NA | + | 6.00 | -24.75 | 62.63 | 32.23 | 0.00 |
| -0.84 | NA | NA | NA | NA | 0.00 | + | NA | 4.00 | -27.07 | 62.67 | 32.27 | 0.00 |
| -18.94 | NA | NA | NA | NA | NA | + | + | 6.00 | -24.80 | 62.72 | 32.32 | 0.00 |
| -0.09 | 0.02 | NA | NA | -0.01 | 0.00 | + | NA | 6.00 | -24.81 | 62.73 | 32.33 | 0.00 |
| -17.60 | 0.00 | NA | 0.02 | NA | NA | NA | + | 6.00 | -25.01 | 63.14 | 32.74 | 0.00 |
| -20.31 | NA | NA | NA | 0.00 | 0.00 | NA | + | 6.00 | -25.04 | 63.19 | 32.80 | 0.00 |
| -20.07 | -0.01 | NA | NA | 0.00 | NA | NA | + | 6.00 | -25.09 | 63.29 | 32.89 | 0.00 |
| 0.14 | 0.01 | NA | 0.02 | NA | NA | + | NA | 5.00 | -26.32 | 63.43 | 33.03 | 0.00 |
| -18.53 | NA | NA | 0.02 | NA | NA | + | + | 7.00 | -24.06 | 63.63 | 33.24 | 0.00 |
| -19.43 | NA | NA | 0.02 | 0.00 | 0.00 | NA | + | 7.00 | -24.13 | 63.77 | 33.37 | 0.00 |
| 0.06 | NA | NA | 0.02 | NA | 0.00 | + | NA | 5.00 | -26.50 | 63.79 | 33.39 | 0.00 |
| 0.82 | 0.02 | NA | 0.02 | -0.01 | 0.00 | + | NA | 7.00 | -24.30 | 64.11 | 33.71 | 0.00 |
| -19.37 | 0.00 | NA | 0.02 | 0.00 | NA | NA | + | 7.00 | -24.32 | 64.14 | 33.75 | 0.00 |
| -19.61 | 0.00 | NA | NA | NA | 0.00 | NA | + | 6.00 | -25.52 | 64.15 | 33.75 | 0.00 |
| -0.75 | 0.01 | NA | NA | NA | 0.00 | + | NA | 5.00 | -26.89 | 64.56 | 34.16 | 0.00 |
| -18.86 | 0.01 | NA | 0.02 | NA | 0.00 | NA | + | 7.00 | -24.53 | 64.57 | 34.18 | 0.00 |
| -19.60 | NA | NA | NA | NA | 0.00 | + | + | 7.00 | -24.54 | 64.59 | 34.19 | 0.00 |
| -19.82 | -0.01 | NA | NA | NA | NA | + | + | 7.00 | -24.57 | 64.66 | 34.26 | 0.00 |
| -19.28 | NA | NA | 0.02 | NA | 0.00 | + | + | 8.00 | -23.59 | 65.15 | 34.75 | 0.00 |
| -19.59 | -0.01 | NA | 0.02 | NA | NA | + | + | 8.00 | -23.65 | 65.26 | 34.87 | 0.00 |
| -20.25 | 0.00 | NA | NA | 0.00 | 0.00 | NA | + | 7.00 | -25.03 | 65.58 | 35.18 | 0.00 |
| 0.21 | 0.01 | NA | 0.02 | NA | 0.00 | + | NA | 6.00 | -26.28 | 65.67 | 35.27 | 0.00 |
| -19.47 | 0.01 | NA | 0.02 | 0.00 | 0.00 | NA | + | 8.00 | -24.03 | 66.03 | 35.63 | 0.00 |
| -19.33 | 0.01 | NA | NA | NA | 0.00 | + | + | 8.00 | -24.52 | 67.02 | 36.63 | 0.00 |
| -19.00 | 0.01 | NA | 0.02 | NA | 0.00 | + | + | 9.00 | -23.57 | 67.65 | 37.25 | 0.00 |
| -3.97 | -0.05 | + | NA | 0.00 | 0.00 | NA | NA | 5.00 | -30.24 | 71.27 | 40.88 | 0.00 |
| -2.90 | -0.04 | + | 0.02 | 0.00 | 0.00 | NA | NA | 6.00 | -29.09 | 71.30 | 40.90 | 0.00 |
| -1.85 | -0.04 | + | 0.02 | NA | 0.00 | NA | NA | 5.00 | -30.72 | 72.23 | 41.83 | 0.00 |
| -2.90 | -0.04 | + | NA | NA | 0.00 | NA | NA | 4.00 | -31.89 | 72.29 | 41.89 | 0.00 |
| -2.23 | -0.03 | + | 0.02 | 0.00 | NA | NA | NA | 5.00 | -31.22 | 73.23 | 42.83 | 0.00 |
| -1.47 | -0.02 | + | 0.02 | NA | NA | NA | NA | 4.00 | -32.38 | 73.28 | 42.88 | 0.00 |
| -3.36 | -0.03 | + | NA | 0.00 | NA | NA | NA | 4.00 | -32.71 | 73.94 | 43.55 | 0.00 |
| -2.58 | -0.02 | + | NA | NA | NA | NA | NA | 3.00 | -33.90 | 74.10 | 43.70 | 0.00 |
| -1.33 | NA | + | 0.03 | NA | NA | NA | NA | 3.00 | -34.97 | 76.25 | 45.86 | 0.00 |
| -1.75 | -0.04 | NA | NA | 0.00 | 0.00 | NA | NA | 4.00 | -34.23 | 76.97 | 46.57 | 0.00 |
| -0.73 | -0.04 | NA | 0.02 | 0.00 | 0.00 | NA | NA | 5.00 | -33.21 | 77.20 | 46.80 | 0.00 |
| -1.04 | -0.03 | NA | NA | NA | 0.00 | NA | NA | 3.00 | -35.50 | 77.30 | 46.91 | 0.00 |
| -0.04 | -0.03 | NA | 0.02 | NA | 0.00 | NA | NA | 4.00 | -34.52 | 77.55 | 47.15 | 0.00 |
| 0.22 | -0.02 | NA | 0.02 | NA | NA | NA | NA | 3.00 | -35.84 | 77.98 | 47.58 | 0.00 |
| -1.57 | NA | + | 0.03 | 0.00 | NA | NA | NA | 4.00 | -34.79 | 78.09 | 47.69 | 0.00 |
| -0.33 | -0.02 | NA | 0.02 | 0.00 | NA | NA | NA | 4.00 | -34.83 | 78.19 | 47.79 | 0.00 |
| -1.31 | NA | + | 0.03 | NA | 0.00 | NA | NA | 4.00 | -34.91 | 78.34 | 47.94 | 0.00 |
| -0.86 | -0.02 | NA | NA | NA | NA | NA | NA | 2.00 | -37.14 | 78.43 | 48.04 | 0.00 |
| -1.42 | -0.03 | NA | NA | 0.00 | NA | NA | NA | 3.00 | -36.18 | 78.66 | 48.26 | 0.00 |
| 0.24 | NA | NA | 0.03 | NA | NA | NA | NA | 2.00 | -37.75 | 79.66 | 49.26 | 0.00 |
| -1.55 | NA | + | 0.03 | 0.00 | 0.00 | NA | NA | 5.00 | -34.72 | 80.23 | 49.83 | 0.00 |
| -3.00 | NA | + | NA | NA | NA | NA | NA | 2.00 | -38.05 | 80.24 | 49.85 | 0.00 |
| -0.06 | NA | NA | 0.03 | 0.00 | NA | NA | NA | 3.00 | -37.43 | 81.17 | 50.78 | 0.00 |
| 0.26 | NA | NA | 0.03 | NA | 0.00 | NA | NA | 3.00 | -37.73 | 81.76 | 51.37 | 0.00 |
| -2.97 | NA | + | NA | NA | 0.00 | NA | NA | 3.00 | -37.97 | 82.26 | 51.86 | 0.00 |
| -3.12 | NA | + | NA | 0.00 | NA | NA | NA | 3.00 | -38.01 | 82.33 | 51.94 | 0.00 |
| -1.42 | NA | NA | NA | NA | NA | NA | NA | 1.00 | -40.47 | 82.99 | 52.60 | 0.00 |
| -0.04 | NA | NA | 0.03 | 0.00 | 0.00 | NA | NA | 4.00 | -37.41 | 83.34 | 52.94 | 0.00 |
| -3.09 | NA | + | NA | 0.00 | 0.00 | NA | NA | 4.00 | -37.94 | 84.40 | 54.00 | 0.00 |
| -1.63 | NA | NA | NA | 0.00 | NA | NA | NA | 2.00 | -40.38 | 84.90 | 54.51 | 0.00 |
| -1.39 | NA | NA | NA | NA | 0.00 | NA | NA | 2.00 | -40.44 | 85.02 | 54.63 | 0.00 |
| -1.60 | NA | NA | NA | 0.00 | 0.00 | NA | NA | 3.00 | -40.34 | 86.99 | 56.59 | 0.00 |
